# IL-4 rs2243250 polymorphism associated with susceptibility to allergic rhinitis: a meta-analysis

**DOI:** 10.1042/BSR20210522

**Published:** 2021-04-22

**Authors:** Feifei Jiang, Aihui Yan

**Affiliations:** Department of Otorhinolaryngology, The First Hospital of China Medical University, Shenyang, Liaoning, China

**Keywords:** Allergic rhinitis, Interleukin-4, Polymorphism

## Abstract

Objective: The relationship between IL-4 rs2243250 polymorphism and the risk of allergic rhinitis is not clear at present. The present study aims to evaluate the exact association between IL-4 rs2243250 polymorphism and susceptibility to allergic rhinitis by a meta-analysis.

Methods: The studies about IL-4 rs2243250 polymorphism associated with susceptibility to allergic rhinitis were searched using PubMed, Excerpta Medica Database (EMBASE), Web of Science, Cochrane Library, China National Knowledge Infrastructure (CNKI) and China Wanfang databases. The last search time was on March 1, 2021. Data analysis was performed using Stata 15.0 software.

Results: Nine documents were enrolled, from which 1709 patients with allergic rhinitis were included. Among them, six genotype frequencies in the control group conformed to Hardy–Weinberg Equilibrium (HWE). The meta-analysis of all included studies showed significant heterogeneity of each gene model. After omitting the studies whose genotype frequency in the control group did not meet the requirements of HWE, no significant heterogeneity was found in each gene model. The meta-analysis results of the control group genotypes in line with the HWE showed statistically significant differences in the pooled odds ratio (OR) of allele model (T vs. C), recessive model (TT vs. TC+CC) and homozygous model (TT vs. CC), which were 1.19 (95%CI: 1.04–1.35), 1.28 (95%CI: 1.06–1.55) and 1.56 (95%CI: 1.13–2.17), respectively. No statistically significant difference was observed in dominant and heterozygous genetic models.

Conclusion: IL-4 rs2243250 single nucleotide polymorphism associated with susceptibility to allergic rhinitis, allele T and genotype TT could increase the risk of allergic rhinitis.

## Introduction

Allergic rhinitis (AR) refers to chronic inflammatory response characterized by the release of IgE-mediated inflammatory mediators, and generation of a large number of multiple immunocompetent cells and cytokines in the nasal mucosa of specific individuals after exposure to allergens [1]. The clinical manifestations of AR include nasal obstruction, water-like nasal discharge, continuous sneezing and nasal itching, which can be cured themselves or ameliorated after treatment. AR is a relatively common disease and its incidence has been increasing rapidly worldwide in the past two decades, reaching 9-40% [2,3]. AR is not a fatal disease, but it is a most common allergic disease that the clinical symptoms will have a serious impact on the life and daily work of patients, causing a huge economic burden to society. In addition, a large number of studies have shown that AR is a risk factor for asthma [4]. The treatment of AR can prevent and control the occurrence of asthma. Therefore, it has been one of the critical diseases in the respiratory system at present, and also the focus and hotspot of medical researches.

Single nucleotide polymorphism (SNP) is the simplest form of gene polymorphism that exists widely in the organisms’ genome. It usually refers to the difference between single nucleotide sequences in individual genomic DNA, which can exist in a certain type of population or normal individuals [5]. Gene polymorphism is a very crucial genetic factor in AR that can up-regulate or down-regulate the susceptibility of AR, which is also one of the main reasons for the clinical diversification of AR, leading to differences between individual treatments [6]. Interleukin 4 (IL-4) is a pleiotropic cytokine produced by activated Th2 cell subsets, B lymphocytes, mast cells, basophils and other inflammatory cells. It is an essential regulator of the Th2 cell immune response [7]. IL-4 can promote humoral immunity and antagonize the activity of Th1 cytokines (IFN, IL-1, etc.) [8]. The serum level of IL-4 is significantly increased in AR [9].

There are multiple polymorphic loci in IL-4, such as the promoter region rs2243250 (C-590T or C-589T), exon 1 region C+33T, T, intron 2 region GT repeat sequence and G +3017T and intron 3 region 70bP variable number of tandem repeats (VNTR). At present, many studies have revealed that IL-4 rs2243250 polymorphism correlates with susceptibility to AR, but their conclusions are not consistent. Some researchers believe that IL-4 rs2243250 allele T will increase the risk of AR [10,11]. Some believe that its allele T will reduce the susceptibility to AR [12]. Others have reported that its allele T is not related to AR [13,14]. Therefore, we systematically collected the literature on the relationship between IL-4 rs2243250 polymorphism and the susceptibility to AR, and performed a meta-analysis to analyze their relationship comprehensively.

## Methods

### Literature retrieval

This meta-analysis was conducted under the guidance of the Preferred Reporting Items for Systematic Reviews and Meta-analysis (PRISMA) [15]. The case–control studies on IL-4 rs2243250 single nucleotide polymorphism associated with susceptibility to AR were searched comprehensively and systematically in PubMed, Excerpta Medica Database (EMBASE), Web of Science, Cochrane Library, China National Knowledge Infrastructure (CNKI) and China Wanfang databases. The last search time was on March 1, 2021. The search strategy was as follows: (‘Allergic rhinitis’ OR ‘rhinitis’ OR ‘Allergic disease’) AND (‘IL-4’ OR ‘Interleukin 4’) AND (‘Polymorphism’ OR ‘single nucleotide mutation’ OR ‘mutation’). We provided a detailed retrieval process in Pubmed (Supplementary Table S1). Besides, we manually searched the articles in the reference lists of all included studies and recently published reviews. If there were several kinds of research from the same test, only the most recent or complete research was included. The language was not restricted. Two researchers searched each database independently, and finally cross-checked. If there were differences, they would be resolved through discussion.

### Inclusion and exclusion criteria

#### Inclusion criteria

(1) The research was designed as a case–control study; (2) enough genotype data were included in calculating the odds ratio (OR) and its 95% confidence interval (CI); (3) IL-4 rs2243250 (C-590T or C-589T) was the gene research site.

#### Exclusion criteria

(1) Animal research or cell research; (2) case reports, editorials and reviews; (3) the Newcastle–Ottawa scale (NOS) score was less than six.

### Quality assessment

The qualities of the literatures were evaluated according to the NOS [16]. Articles with no more than six stars were of low quality, while those with six stars or more were of high quality. The published studies assessed with six stars or more were enrolled in this meta-analysis.

### Data extraction

According to the pre-made form, the data in the literature were independently extracted, cross-checked by two researchers. For each included study, we collected the following information: first author, publication year, region, ethnicity, detecting method, genotype frequency of IL-4 rs2243250 in patients with AR and controls.

### Statistical analysis

Data analysis was performed using Stata 15.0 statistic software. The OR and its 95% CI were selected as the effect size to estimate the distribution of genotypes in the allele model (T vs. C) and dominant genetic model (TT + TC vs. CC), recessive genetic model (TT vs. TC + CC), homozygous genetic model (TT vs. CC) and heterozygous genetic model (TC vs. CC) of the AR group and the control group. The Hardy–Weinberg equilibrium (HWE) test was performed on the genotype distribution of the control group. The heterogeneity test was conducted using *Q*-test and *I*^2^ statistics. If there was heterogeneity (*P*≤0.05 or *I*^2^≥50%), a random-effects model (REM) was used; otherwise, a fixed-effects model (FEM) was applied. The heterogeneity across the studies existed, subgroup analysis conducted based on Ethnicity, mutation site (C-590T or C-589T) and HWE. The publication bias was judged intuitively by drawing a funnel plot. The funnel plot symmetric, no publication bias was found; otherwise, publication bias existed. Besides, publication bias was quantitatively evaluated by the Egger’s Test. Finally, a sensitivity analysis was performed to verify the robustness of the obtained findings.

## Results

### Literature search results

**Figure 1:**
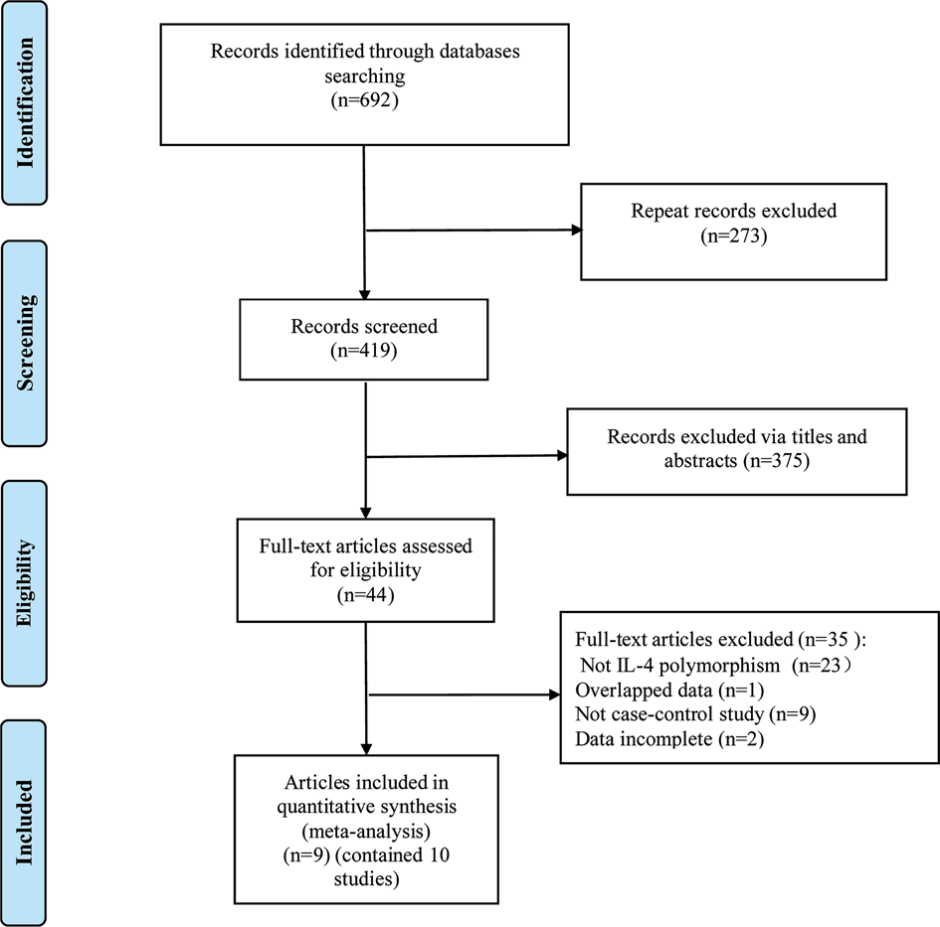
Flow diagram of document retrieval

After rigorous screening, we finally identified that nine articles met the standard [10–14,17–20], including 1709 patients with AR. The detailed literature screening process is shown in Figure 1. All included articles are published in English. The basic characteristics of the included articles and the NOS score of articles are shown in Table 1. The NOS of all studies were above six stars of high-quality.

**Table 1:** The basic characteristics of the studied included on the relationship between IL-4 rs2243250 polymorphism and susceptibility to allergic rhinitis

Study	Year	Country	Gene site	Detection method	Allergic rhinitis			Control			HWE	NOS
					TT	TC	CC	TT	TC	CC		
Valatabar a [14]	2020	Iran	C-590T	PCR-RFLP	12	101	137	15	37	98	0.000	8
Valatabar b [14]	2020	Iran	C-590T	PCR-RFLP	19	53	178	4	41	105	0.999	8
Al-Rawashdeh [20]	2020	Jordan	C-589T	PCR-RFLP	9	53	96	2	50	88	0.082	8
Shirkani [11]	2019	Iran	C-590T	PCR-RFLP	47	31	8	0	52	34	0.000	7
Dahmani [13]	2016	Algeria	C-589T	PCR-RFLP	9	32	6	10	12	11	0.118	8
Micheal [19]	2013	Pakistan	C-589T	PCR-RFLP	19	63	26	5	84	31	0.000	7
Movahedi [12]	2013	Iran	C-590T	PCR-SSP	0	63	35	0	129	10	0.000	7
Miyake [17]	2012	Japan	C-590T	TaqMan SGA	172	180	41	286	330	87	0.585	8
Yadav [18]	2012	Malaysia	C-589T	PCR-RFLP	29	17	8	24	14	6	0.118	8
Lu MP [10]	2011	China	C-590T	TaqMan SGA	179	81	5	157	107	11	0.166	8

Abbreviations: HWE, Hardy–Weinberg equilibrium; NOS, Newcastle–Ottawa scale; PCR, polymerase chain reaction; RFLP, restriction fragment length polymorphism; SGA, SNP genotyping assay; SSP, sequence specific-primers.

### Meta-analysis results

#### Allele comparison

**Figure 2:**
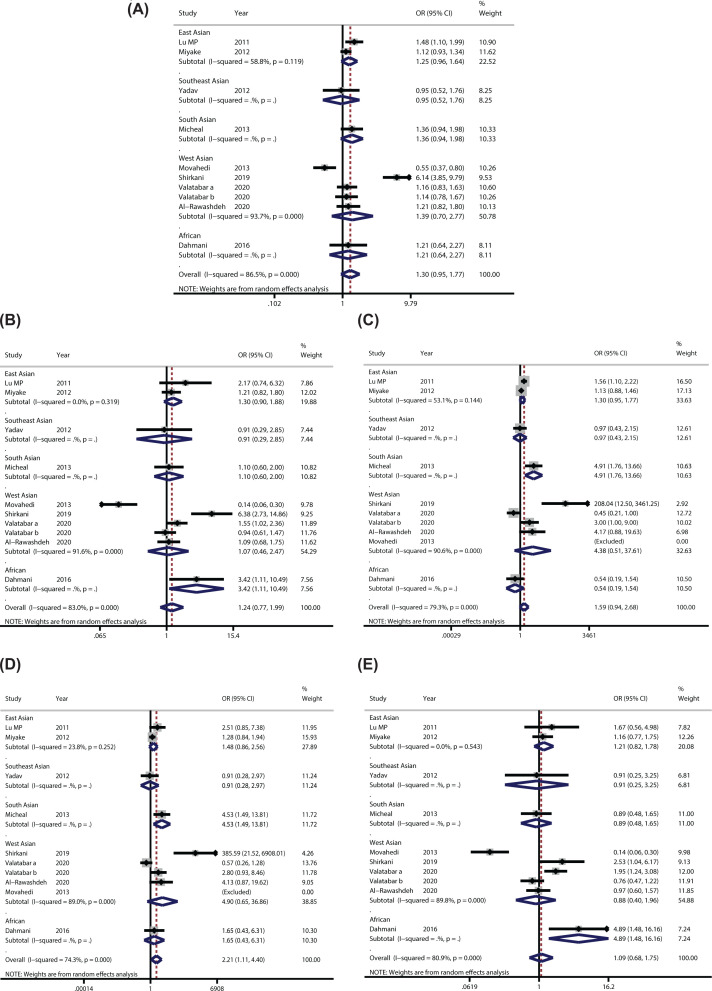
Forest plots of the relationship between IL-4 rs2243250 polymorphism and susceptibility to allergic rhinitis (**A**) Allele model; (**B**) Dominant model; (**C**) Recessive model; (**D**) Homozygous model; (**E**) Heterozygous model.

**Figure 3:**
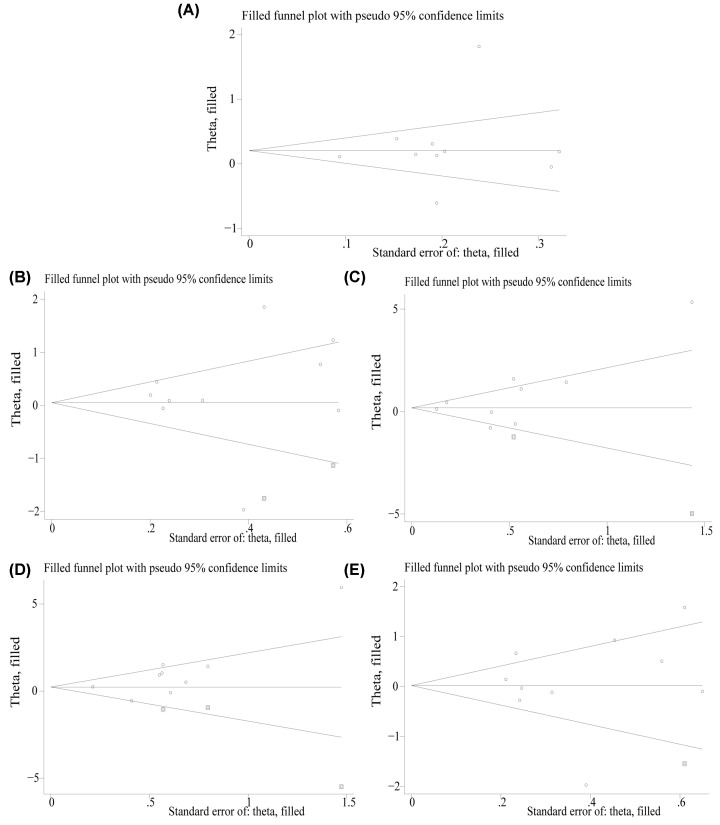
Funnel plots of the relationship between IL-4 rs2243250 polymorphism and susceptibility to allergic rhinitis (**A**) Allele model; (**B**) Dominant model; (**C**) Recessive model; (**D**) Homozygous model; (**E**) Heterozygous model.

The meta-analysis results of the association between IL-4 rs2243250 single nucleotide polymorphism and susceptibility to AR were shown in Table 2. As seen from the results of heterogeneity analysis, *I*^2^=86.5% (*P*<0.05), so a REM was used. Compared with the control group, no significant difference was found in IL-4 rs2243250 allele T relative to allele C in the AR group (pooled OR = 1.3; 95%CI: 0.95–1.77, *P*>0.05). After subgroup analysis based on Ethnicity, the difference was not statistically significant, and the heterogeneity was not decreased significantly (Figure 2A). According to the subgroup analysis of gene locus, no difference was detected between IL-4 C-590T and C-589T alleles. The subgroup analysis results of genotype frequency in the control group conformed to the HWE showed that statistically significant difference, but no significant heterogeneity was found (OR = 1.19; 95%CI: 1.04–1.35, *P*<0.05). Egger’s test showed *P*>0.05, while the symmetry of the funnel plot was general (Figure 3A), indicating a certain publication bias.

**Table 2:** Main results of meta-analysis of IL-4 RS2243250 polymorphism and susceptibility to allergic rhinitis

Genetic model	Subgroup	*n*	OR	95%CI	*P* for OR	*I*^2^(%)	*P* for heterogeneity	Model	*P* for Egger
Allelic model (T vs. C)	Overall	9	1.3	0.95–1.77	0.105	86.5	0.000	REM	0.447
	C-590T	5	1.39	0.86–2.20	0.188	92.4	0.000	REM	0.331
	C-589T	4	1.23	0.97–1.54	0.084	0.0	0.809	FEM	0.254
	HWE (yes)	6	1.19	1.04–1.35	**0.01**	0.0	0.697	FEM	0.930
Dominant model (TT+TC vs. CC)	Overall	9	1.24	0.77–1.99	0.375	83.0	0.000	REM	0.246
	C-590T	5	1.2	0.58–2.48	0.625	89.8	0.000	REM	0.357
	C-589T	4	1.22	0.82–1.83	0.327	21.2	0.283	FEM	0.413
	HWE (yes)	6	1.18	0.93–1.48	0.201	16.4	0.308	FEM	0.151
Recessive model (TT vs. TC+CC)	Overall	9	1.59	0.94–2.68	0.085	79.3	0.000	REM	0.177
	C-590T	5	1.55	0.78–3.08	0.206	84.9	0.000	REM	0.207
	C-589T	4	1.69	0.59–4.84	0.325	74.5	0.008	REM	0.478
	HWE (yes)	6	1.28	1.06–1.55	**0.009**	48.8	0.082	FEM	0.284
Homozygous model (TT vs. CC)	Overall	9	2.21	1.11–4.40	**0.024**	74.3	0.000	REM	0.149
	C-590T	5	2.41	0.83–6.98	0.106	84.2	0.000	REM	0.188
	C-589T	4	2.25	1.02–4.97	**0.045**	34.3	0.207	FEM	0.994
	HWE (yes)	6	1.56	1.13–2.17	**0.007**	0.5	0.413	FEM	0.137
Heterozygote model (TC vs. CC)	Overall	9	1.09	0.68–1.75	0.718	80.9	0.000	REM	0.296
	C-590T	5	0.99	0.49–1.99	0.982	87.6	0.000	REM	0.818
	C-589T	4	1.22	0.67–2.22	0.511	55.5	0.081	REM	0.375
	HWE (yes)	6	1.07	0.84–1.36	0.590	45.3	0.103	FEM	0.207

Abbreviations: FEM, fixed-effect model; OR, odds ratio; REM, random-effect model.

#### Comparison of recessive genetic model

As depicted in Table 2, in the recessive genetic model (TT vs. TC+CC), the heterogeneity was high (*I*^2^=79.3%), a REM used for data analysis. The results showed no significant difference (OR = 1.59; 95%CI: 0.94–2.68, *P*>0.05). After subgroup analysis based on Ethnicity, significant heterogeneity was observed without statistically significant difference (Figure 2C). According to IL-4 gene locus (IL-4 C-590T and C-589T) subgroup analysis, the recessive models of the two were not significant. There were six documents in which genotype frequency in the control group accorded with HWE. The results of the FEM showed that the difference was statistically significant, but the heterogeneity among the studies was significantly reduced (OR = 1.28; 95%CI: 1.06–1.55, *P*<0.05). The Egger's test showed *P*>0.05. The funnel plot was symmetrical (Figure 3C), indicating that publication bias was well controlled.

#### Comparison of homozygous genetic model

In the homozygous genetic model (TT vs. CC), the heterogeneity across the studies was significant (*I*^2^=74.3%). The results of the REM showed statistically significant difference (OR = 2.21; 95%CI: 1.11–4.40, *P*<0.05), (Figure 2D). After subgroup analysis based on ethnicity, the heterogeneity declined significantly. As shown in Table 2, the difference was not statistically significant regarding the IL-4 C-590T locus, while statistically significant was detected regarding the IL-4 C-589T locus (OR = 2.25; 95%CI: 1.02–4.97, *P*<0.05). The subgroup analysis of genotype frequency in the control group meeting the HWE revealed that the difference was statistically significant between the articles without significant heterogeneity (OR = 1.56; 95%CI: 1.13–2.17, *P*<0.05). The funnel plot was symmetrical (Figure 3D). Egger’s test results showed *P*>0.05, which indicated no significant publication bias.

#### Comparison of dominant and heterozygous genetic models

The forest plots of the dominant (TT + TC vs*.* CC) and heterozygous (TC vs. CC) genetic models were shown in Figure 2B,E, respectively. The funnel plots were shown in Figure 3B,E, respectively. Either overall comparison or subgroup analyses based on ethnicity and gene locus, no difference was found in genotype in these two genetic models (Table 2). Through the funnel plot and Egger’s test, none of them showed significant publication bias.

### Sensitivity analysis

**Figure 4:**
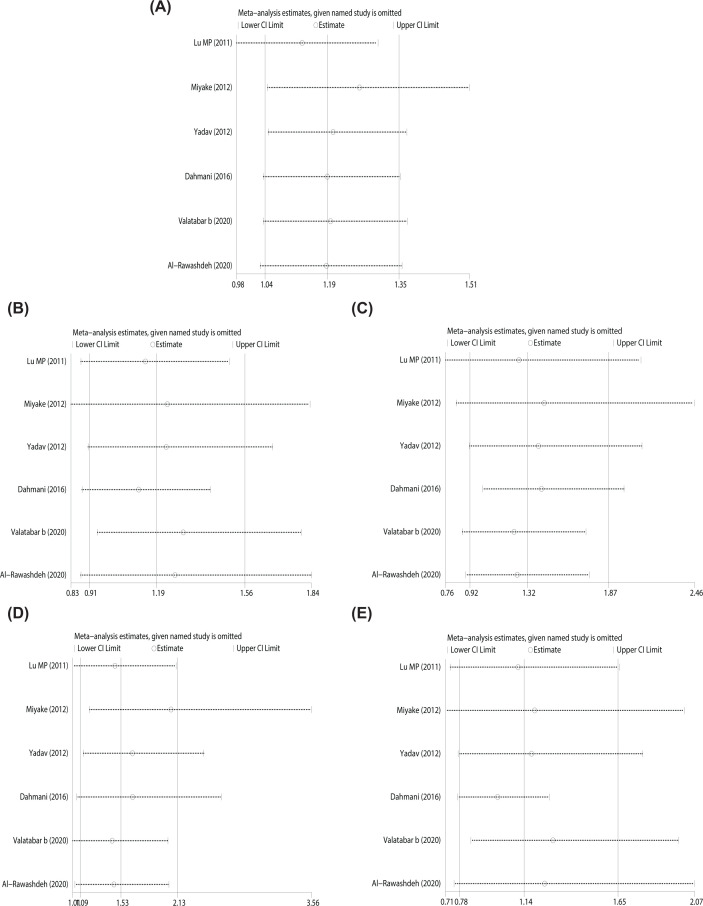
Sensitivity analysis of subgroups with genotype frequency meeting HWE in the control group (**A**) Allele model; (**B**) Dominant model; (**C**) Recessive model; (**D**) Homozygous model; (**E**) Heterozygous model; HWE, Hardy–Weinberg equilibrium.

As HWE had a greater impact on heterogeneity, we only conducted a sensitivity analysis for the articles where genotype frequency in the control group confirmed to HWE. After omitting each article, we re-conducted the meta-analysis to explore the impact of a certain study on the overall results. In the allele model (Figure 4A) and recessive genetic model (Figure 4C), after excluding the study of Lu et al. [10], the difference was not statistically significant. Nevertheless, in the dominant (Figure 4B), homozygous (Figure 4D) and heterozygous (Figure 4E) models, no changes inconsistent with the original conclusion occurred after any study was omitting. This showed that allelic models and recessive gene models had a certain degree of instability. In the dominant, homozygous and heterozygous models, the conclusions had good robustness.

## Discussion

To explore the relationship between IL-4 rs2243250 polymorphism and susceptibility to AR, we conducted this meta-analysis. The results of our study confirmed a significant correlation between IL-4 rs2243250 polymorphism and the risk of AR. A variety of cytokines play a critical role in the occurrence and development of allergic diseases. Most of the researches on the genomics of AR are related to the cytokines and coding genes of their corresponding receptors. The interaction between IL-4 and its corresponding receptor crucially functions in the pathological process of AR and asthma. At present, a prior study has demonstrated a close association of IL-4 and its receptor gene polymorphisms with the pathogenesis of allergic diseases [21]. It is well known that IL-4 plays a key role in the pathogenesis of AR, and excessive secretion of IL-4 can cause allergic reactions. The study reported by Li et al. has shown that in allergic diseases, serum IL-4 level is significantly increased, and IL-4 rs2243250 polymorphism is significantly related to allergic diseases [22]. The level of IL-4 *in vivo* is regulated by the IL-4 precursor region at the transcription level. Under the stimulation of certain pathogenic factors, T helper 2 (Th2) cells will induce the synthesis of the IL-4 gene and release IL-4. In addition, individual genetic factors will also affect the level of IL-4 in serum. Serum IgE level is used as a main long-term indicator to assess the severity of allergic diseases. Robinson et al. have reported that mice with impaired IL-4 haplotypes have significantly impaired specific IgE response to allergens [23].

After a systematic retrieval and strict inclusion, nine available studies published in English were finally included in our meta-analysis. According to the quality assessment, the NOS scores of all studies were six stars above, suggesting that the quality of the included studies was high. The results showed significant heterogeneity in the allele, dominant, recessive, homozygous and heterozygous genetic models. Among them, the difference is statistically significant only in the homozygous genetic model. Except for the allele model, the funnel plots of other gene models were generally symmetrical, while Egger’s test showed the *P*-values all higher than 0.05, indicating that the studies included in our meta-analysis had no significant publication bias. According to subgroup analyses based on Ethnicity, the heterogeneity of the genetic models of each gene did not decrease significantly. Hence, ethnicity is not the main source of heterogeneity. On the basis of the subgroup analysis of the IL-4 rs2243250 locus, it is basically the same as the overall analysis. However, heterogeneity was decreased significantly regarding the IL-4 C-589T locus in the allele, dominant, heterozygous and homozygous genetic models, so gene locus is also an important source of heterogeneity.

At the same time, we also observed that after excluding the study with genotype frequency in the control group not conformed to the HWE, the heterogeneity in each gene model of the IL-4 rs2243250 locus was significantly reduced. This showed that HWE was the main source of heterogeneity. The meta-analysis of the six studies in which the genotype frequency in the control group meeting the HWE showed that, except the homozygous, dominant and heterozygous genetic models that showed consistent conclusions in the overall analysis, allele and recessive genetic models showed statistical significance. In other words, in the IL-4 rs2243250 locus, allele T and genotype TT will increase the risk of AR. Sensitivity analysis confirmed the robustness of the conclusions in the homozygous genetic model, but the allele and recessive gene models still had certain instabilities. Therefore, the conclusion that the allele T increases the susceptibility to AR is still to be cautiously considered.

Inevitably, the present study also had certain limitations. First, this meta-analysis only included publicly published articles, yet the unpublished potentially high-quality studies are not included. This may cause publication bias to a certain extent. Second, few studies were included in the analysis and the sample size was not large, which might have a certain impact on the robustness of the conclusion. Third, the scope of the included studies was relatively limited. Most of the articles were from Asian countries, with only one from African countries and no article from European and American countries. Fourth, sensitivity analysis showed a certain degree of instability in the allele and recessive gene models. Fifth, the influence of IL-4 combined with other genes (such as IL-6, IL-10, IL-17, IL-27, IFN-γ, etc.) or the environments on allergies has not been analyzed.

In conclusion, the meta-analysis suggests a significant correlation between IL-4 rs2243250 single nucleotide polymorphism and susceptibility to AR and that allele T and genotype TT could increase the risk of AR. Meanwhile, we have also noticed that this study still has certain limitations, such as sample size and gene-gene interaction. Therefore, more and more in-depth studies on a larger scale are necessary to be performed in the future to explore the correlation between the IL-4 rs2243250 polymorphism and the susceptibility to AR.

## Supplementary Material

Supplementary Table S1

## Data Availability

The datasets used and/or analysed during the current study are available from the corresponding author on reasonable request.

## Abbreviations

AR: allergic rhinitis
CI: confidence interval
FEM: fixed-effects model
HWE: Hardy–Weinberg equilibrium
IL-4: interleukin 4
NOS: Newcastle–Ottawa scale
OR: odds ratio
PRISMA: Preferred Reporting Items for Systematic Reviews and Meta-analysis
REM: random-effects model
SNP: single nucleotide polymorphism
VNTR: variable number of tandem repeats
