# Supplementary material for: IL-4 rs2243250 polymorphism associated with susceptibility to allergic rhinitis: a meta-analysis
Source: Biosci Rep. 2021 Apr 22;41(4):BSR20210522. doi: 10.1042/BSR20210522 (PMC8065178; doi:10.1042/BSR20210522)
Supplement: Supplementary Table S1 [file BSR-2021-0522_supp.pdf]

Supplementary Table 1. Retrieval steps and results in PubMed (The retrieval time:20210301).

Search	Query	Items found
#1	Allergic rhinitis[MeSH terms]	21187
#2	"Allergic rhinitis"[Title/Abstract] OR "rhinitis"[Title/Abstract] OR “Allergic disease”[All fields]	80603
#3	#1 OR #2	86916
#4	Interleukin 4[MeSH terms]	23038
#5	“Interleukin 4”[Title/Abstract] OR "IL-4"[Title/Abstract]	49696
#6	#4 OR #5	53527
#7	Polymorphism [MeSH terms]	279727
#8	“Polymorphism”[Title/Abstract] OR “single nucleotide mutation”[Title/Abstract] OR “mutation”[Title/Abstract]	541705
#9	#7 OR #8	679051
#10	#3 AND #6 AND #9	141
